# Response of Maize Yield and Nitrogen Use Efficiency to Integrated Cover Crop Rotation and Nitrogen Management Practices

**DOI:** 10.3390/plants15060877

**Published:** 2026-03-12

**Authors:** Wei Qi, Long Zhang, Qila Sa, Wenhua Xu, Yanjie Lv, Shan Lan, Fanyun Yao, Yongjun Wang

**Affiliations:** 1 College of Agronomy, Jilin Agricultural University, 2888 Xincheng St., Changchun 130118, China; 4368731@163.com (W.Q.); zhanglong_1197@163.com (L.Z.); qilasa@163.com (Q.S.); 2 Key Laboratory of Crop Ecophysiology and Farming System in Northeast China, Ministry of Agriculture and Rural Affairs, Institute of Agricultural Resource and Environment, Jilin Academy of Agricultural Sciences, 1363 Shengtai St., Changchun 130033, China; ecowhxu@126.com (W.X.); lvyanjie_1977@163.com (Y.L.); 3 Guizhou Phosphate (Group) Co., Ltd., Guiyang 550005, China; 13041128733@163.com; 4 Wengfu (Group) Co., Ltd., Guiyang 550005, China; 5 College of Plant Science, Jilin University, 5333 Xi’an Ave, Changchun 130062, China

**Keywords:** rotational cover cropping, field experiment, net assimilation rate, maize production, principal component analysis

## Abstract

Rotational cover cropping is a key practice in conservation agriculture. To investigate the effects of maize-crop rotation with cover crops combined with nitrogen management on maize yield, nitrogen use efficiency (NUE), and related traits, a field experiment was conducted from 2023 to 2025. The experiment employed a split-plot design. The main plots consisted of three cropping systems: continuous maize (Fumin 985’) monoculture (CK), maize rotated with rapeseed (CC-Ra), and maize rotated with rye (CC-Ry). The subplots comprised five nitrogen (N) fertilizer application rates (0, 75, 150, 225, and 300 kg ha^−1^) respectively. Compared to CK, CC-Ra and CC-Ry increased average maize grain yield by 5.93% and 12.89%, and NUE by 8.09% and 2.89%, respectively. At the silking stage, these treatments increased average DM by 6.45% and 16.55%, respectively, and by 5.75% and 15.01% at the maturity stage. The maximum LAI was enhanced by an average of 16.24% and 26.82%, while the net photosynthetic rate (Pn) of the ear leaf increased by 12.29% and 26.32%, respectively. In contrast, the leaf net assimilation rate (NAR) decreased by an average of 19.98% and 18.01%. While higher N application boosted yield, it sharply reduced NUE. Notably, yields under rotations at 225 kg N ha^−1^ matched the yield of continuous maize at 300 kg N ha^−1^. This suggests that the inclusion of cover crops can substitute for a portion of nitrogen fertilizer input while maintaining stable maize yield. Principal component analysis fundamentally clarified that maize rotational cover cropping combined with nitrogen fertilizer management significantly promotes yield. While cover crops increase maize yield, they also facilitate nitrogen accumulation and enhance NUE, albeit at the expense of leaf net assimilation rate. Therefore, balancing the source–sink characteristics of the maize population is necessary to avoid the loss of advantages conferred by rotational cover cropping. This study holds significant implications for incorporating cover crops into maize production systems.

## 1. Introduction

Fertilizers, as a critical agricultural input for enhancing crop productivity, have played a vital role in supporting the sustained growth of grain production in China [1]. However, in recent years, issues such as declining fertilizer use efficiency and degradation of soil functions have become prevalent in the black soil region of Northeast China, largely due to excessive fertilizer application and poor nutrient management practices [2,3]. Concurrently, this black soil region is a major maize (*Zea mays* L.) production area in China, contributing approximately 36% of the national total maize output [4]. Yet the current high yield levels are maintained through substantial fertilizer inputs [5]. This intensive and often inefficient use not only increases management costs but also leads to detrimental effects, including the decline of soil fertility, depletion of soil organic matter (SOM), and disruption of agroecosystem health [6]. Therefore, sustainable agricultural development requires maintaining crop yields while reducing fertilizer inputs.

Nitrogen plays a pivotal role in crop growth and the attainment of high yields [7]. Within an appropriate application range, increasing nitrogen input effectively enhances the leaf area index (LAI) of maize, promotes photosynthetic activity, boosts biomass accumulation, and consequently increases grain yield [8]. However, in current agricultural practice, the blind pursuit of high yields has led to the widespread issue of excessive nitrogen fertilizer application [9]. For instance, the average nitrogen application rate for maize in China is 263 kg N ha^−1^ [10], which is nearly double the crop’s nitrogen requirement. Nitrogen use efficiency (NUE) serves as a crucial indicator for assessing how effectively crops utilize applied nitrogen [11]. Moderately reducing nitrogen application not only improves NUE and minimizes nitrogen losses but can also mitigate environmental pollution by fostering various nitrogen transformation pathways that contribute to O_2_ generation [12,13]. It is noteworthy that excessive nitrogen application increases production costs and poses threats to ecosystem stability and human health, while also exacerbating climate change [14]. Relevant studies indicate that, compared to conventional nitrogen application, nitrogen reduction treatments can lead to a 7.06% decrease in crop yield [15]. In summary, merely increasing or decreasing nitrogen fertilizer dosage struggles to achieve the dual objectives of high yield and enhanced NUE simultaneously. Therefore, there is an urgent need to develop agronomic practices that can substitute or partially replace synthetic nitrogen fertilizers, aiming to improve soil quality and harmonize maize productivity with ecological health.

Cover crops are plants that grow during gaps in the growing season or after the harvest of the main crop, covering the soil either temporally or spatially [16]. They provide multiple benefits, including reducing soil erosion, improving soil fertility, and suppressing weeds [17], and can also significantly increase maize yields. Meta-analysis of existing studies has shown that the cultivation of cover crops can increase maize yields by 7–33% by boosting soil organic carbon sequestration, enhancing weed suppression, and improving soil microbial activity, with the yield increase in mixed cover crop systems reaching 22–30% [18]. Cover crops are generally classified into leguminous (e.g., *Astragalus sinicus* L. and *Trifolium* spp.) and non-leguminous (e.g., *Lolium perenne* L. and *Brassica napus* L.) types [19]. Non-leguminous cover crops are particularly effective at scavenging residual N after main crop harvest [20], and they can enhance maize yield, improve N uptake, and suppress weeds [21]. Moreover, combining cover crops with optimized N management provides an effective approach to maintain or increase crop yields while improving soil quality [14].

In this study, we investigated the combined effects of cover crop rotation and N fertilizer application on maize yield and related physiological processes. The objective of this study is to evaluate the effects of cover crops on maize performance under different nitrogen application rates. Based on the above background, we addressed the following scientific questions: (1) Can cover crop rotation fully or partially substitute N fertilizer while maintaining stable maize grain yield? (2) Can cover crop rotation combined with N management improve yield by optimizing crop population structure? (3) Can cover crop rotation improve maize NUE under different N management regimes? Answering these questions will provide a theoretical basis for the sustainable management of agroecosystems in the black soil region of China.

## 2. Results

### 2.1. Grain Yield and Yield Components

**Figure 1:**
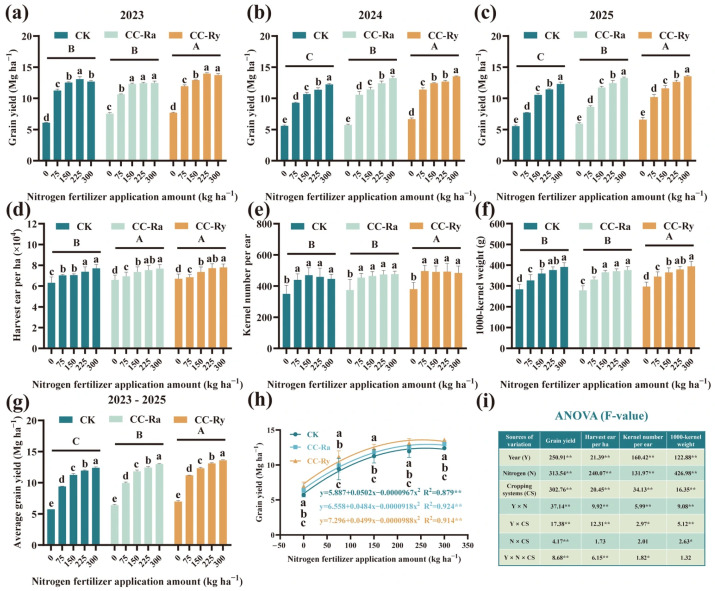
Yield and yield components of different cropping systems under different N conditions during 2023–2025; (**a**–**c**) represent the grain yield in 2023, 2024, and 2025, respectively; (**d**–**f**) represent the harvest ear per ha, the kernel number per ear, and 1000-kernel weight, respectively; (**g**) represents the average grain yield during 2023–2025; (**h**) represents the impact of N fertilizer application amount on grain yield under different treatments; (**i**) represents a three-way ANOVA (F-value). The different lowercase letters in the same group showed a significant difference between each N fertilizer application amount at *p* < 0.05. The different uppercase letters showed the significance between each treatment at *p* < 0.05. * Significant at *p* < 0.05; ** Significant at *p* < 0.01 (LSD). CK: maize monocropping; CC-Ra: maize and rapeseed intercropping; CC-Ry: maize and cereal rye intercropping.

Grain yield of maize was significantly affected by year, nitrogen (N) rate, cover crop treatment, and their interactions. In addition, yield components were also significantly influenced by year, N rate, and cover crop treatment (Figure 1i). Although the average maize yield differed significantly between 2023 and 2025, the effects of cover crops and N rates on yield were consistent across all three years. From 2023 to 2025, compared with the CK treatment, the CC-Ry treatment significantly increased grain yield by an average of 8.42%, 15.35%, and 14.90%, respectively. In 2023, there was no significant difference in yield between CC-Ra and CK; however, in 2024 and 2025, CC-Ra increased grain yield by an average of 8.64% and 9.50% compared with CK (Figure 1a–c). In each year of the experiment, grain yield increased significantly with increasing N rate. Compared with the N0 treatment, the N_75_, N_150_, N_225_, and N_300_ treatments increased grain yield by an average of 59.87%, 85.23%, 96.14%, and 104.09%, respectively. Moreover, the magnitude of yield increase by cover crops varied significantly under different N gradients. Under N_0_ conditions, CC-Ra and CC-Ry increased grain yield by an average of 11.73% and 21.74% compared with CK; under N_75_, the increases were 5.72% and 18.84%; under N_150_, 5.06% and 9.63%; under N_225_, 4.03% and 9.63%; and under N300, 4.81% and 9.67% (Figure 1g). Overall, compared with CK, CC-Ra and CC-Ry treatments significantly increased the number of harvested ears by an average of 2.71% and 2.50%, respectively. CC-Ry also significantly increased kernel number per ear and 1000-kernel weight by 8.39% and 2.47%, respectively (Figure 1d–f). Compared with N_0_, the N_75_, N_150_, N_225_, and N_300_ treatments increased the number of harvested ears by an average of 7.63%, 12.45%, 15.64%, and 18.44%; kernel number per ear by 25.97%, 29.07%, 29.11%, and 27.42%; and 1000-kernel weight by 16.53%, 26.89%, 31.06%, and 35.20%, respectively.

### 2.2. Nitrogen and Dry Matter Accumulation

**Figure 2:**
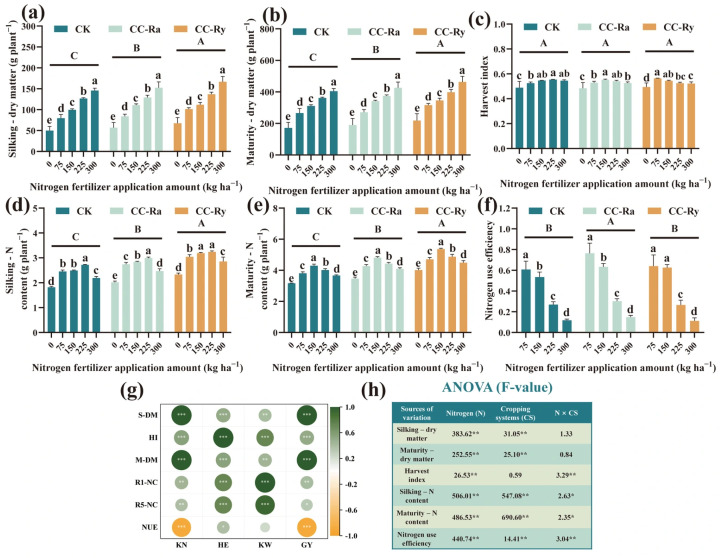
Maize dry matter and N accumulation under different cropping systems and various N fertilizer application amounts in 2025; (**a**) represents the dry matter at the silking period; (**b**) represent the dry matter at the maturity period; (**c**) represent the impact of N fertilizer application amount on harvest index under different treatments; (**d**) represent the N accumulation at silking period; (**e**) represent the N accumulation at maturity period; (**f**) represent the impact of N fertilizer application amount on NUE under different treatments; (**g**) represent the correlation analysis between those index and yield component; (**h**) represent a two-way ANOVA (F-value). The different lowercase letters in the same group showed a significant difference between each N fertilizer application amount at *p* < 0.05. The different uppercase letters showed the significance between each treatment at *p* < 0.05. * Significant at *p* < 0.05. ** Significant at *p* < 0.01. *** Significant at *p* < 0.001 (LSD). CK: maize monocropping; CC-Ra: maize and rapeseed intercropping; CC-Ry: maize and cereal rye intercropping.

Cover crop and nitrogen (N) rate significantly affected dry matter (DM) and N accumulation at both silking and maturity stages, and also strongly regulated NUE (Figure 2h). Compared with the CK treatment, CC-Ra significantly increased DM accumulation at silking and maturity by an average of 6.45% and 5.75%, respectively, while CC-Ry increased these values by 16.55% and 15.01%. Relative to the N_0_ treatment, N_75_, N_150_, N_225_, and N_300_ significantly increased DM accumulation at silking by an average of 52.63%, 86.88%, 128.22%, and 168.99%, respectively (Figure 2a), and at maturity by 47.70%, 73.33%, 97.11%, and 124.16%, respectively (Figure 2b). The harvest index (HI) was only significantly affected by N rate; compared with N_0_, increasing N application increased HI by an average of 10.37% (Figure 2c).

Compared with the CK treatment, CC-Ra significantly increased N concentration at silking and maturity by an average of 11.89% and 11.17%, respectively, while CC-Ry increased these values by 25.72% and 24.02%. Relative to the N_0_ treatment, N_75_, N_150_, N_225_, and N_300_ significantly increased N accumulation at silking by an average of 33.66%, 38.01%, 45.65%, and 21.44%, respectively (Figure 2d), and at maturity by 21.09%, 36.43%, 25.61%, and 15.44%, respectively (Figure 2e). Cover crop rotation improved NUE, with CC-Ra exhibiting the highest overall NUE among the three systems, averaging 46.35% (Figure 2f). NUE decreased significantly with increasing N rate, with average values of 67.18%, 59.97%, 27.92%, and 12.59% under N_75_, N_150_, N_225_, and N_300_ treatments, respectively.

Correlation analysis showed that DM at silking and maturity, nitrogen accumulation at silking and maturity, and harvest index were all significantly and positively correlated with grain yield and its components (Figure 2g). However, NUE was significantly and negatively correlated with both grain yield and kernel number per ear. These results indicate that although increasing N application promotes yield improvement, it simultaneously reduces NUE.

### 2.3. Leaf Area Index (LAI) and Net Assimilation Rate

**Figure 3:**
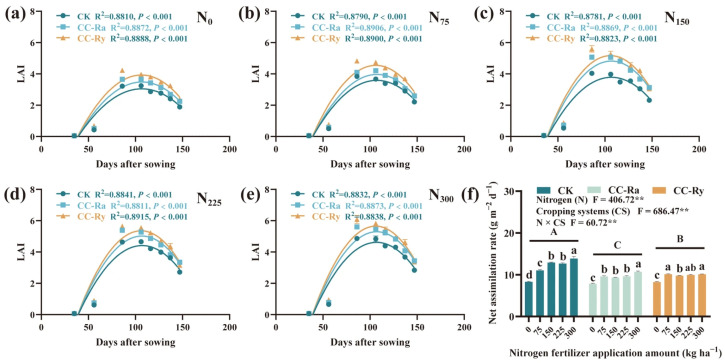
Maize leaf area index (LAI) and net assimilation rate (NAR) of different cropping systems under different N conditions in 2025; (**a**–**e**) represent LAI under different treatments of N_0_, N_75_, N_150_, N_225_ and N_300_, respectively; (**f**) represent the impact of N fertilizer application amount on NAR (R1–R6) under different treatments. The different lowercase letters in the same group showed a significant difference between each N fertilizer application amount at *p* < 0.05. The different uppercase letters showed the significance between each treatment at *p* < 0.05. ** Significant at *p* < 0.01 (LSD).

Under different N rates, the LAI of all treatments followed a quadratic distribution with days after sowing (Figure 3a–e). On average, the maximum LAI of the CC-Ry treatment was higher than that of CC-Ra and CK. The maximum LAI values for CK, CC-Ra, and CC-Ry under N_0_ were 3.04, 3.48, and 3.93, respectively; under N_75_, they were 3.59, 3.97, and 4.55; under N_150_, they were 3.79, 4.82, and 5.16; under N_225_, they were 4.41, 5.02, and 5.35; and under N_300_, they were 4.61, 5.30, and 5.66. In addition, LAI increased with increasing N rate across all three systems. Compared with N_0_, the average LAI of CK under N_75_, N_150_, N_225_, and N_300_ increased by 18.31%, 24.44%, 45.27%, and 51.80%, respectively; for CC-Ra, the increases were 14.23%, 38.24%, 44.44%, and 52.35%; and for CC-Ry, the increases were 15.02%, 30.59%, 35.41%, and 42.99%. Cover crop rotation decreased the net assimilation rate (NAR) of leaves. Compared with CK, CC-Ra and CC-Ry significantly reduced NAR by an average of 19.98% and 18.01%, respectively. Nitrogen application improved NAR; for each 1 kg ha^−1^ increase in N rate, the NAR of CK, CC-Ra, and CC-Ry increased by an average of 0.0170, 0.0077, and 0.0047 g m^−2^ d^−1^, respectively.

### 2.4. Gas Exchange Parameters

**Figure 4:**
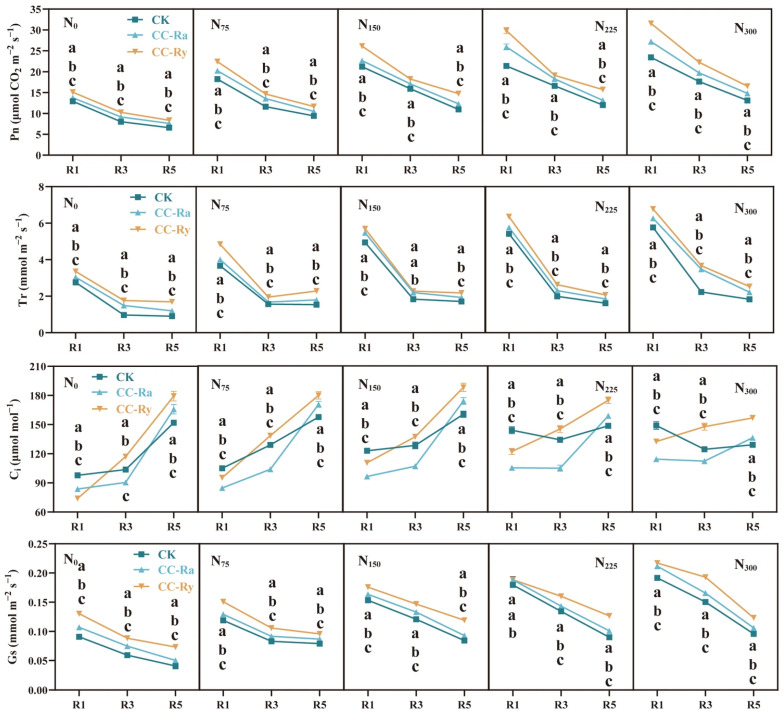
Different cover crop treatments on net photosynthetic rate (P_n_), transpiration rate (T_r_), intercellular CO_2_ concentration (C_i_), and stomatal conductance (G_s_) under various N fertilizer application amounts. The different lowercase letters in the same column showed a significant difference between cropping systems at *p* < 0.05 (LSD test).

The net photosynthetic rate (P_n_), transpiration rate (T_r_), and stomatal conductance (G_s_) reached their maximum values at the silking stage (R1) and gradually decreased during the grain-filling period (Figure 4). Both cover crop rotation and N rate significantly affected these photosynthetic parameters (Table 1). Overall, compared with the CK treatment, CC-Ra and CC-Ry increased P_n_ by an average of 12.29% and 26.32%, T_r_ by 15.35% and 29.31%, and G_s_ by 10.37% and 25.31%, respectively. Relative to the N_0_ treatment, N_75_, N_150_, N_225_, and N_300_ increased P_n_ by an average of 44.11%, 73.50%, 87.49%, and 102.39%, respectively; T_r_ by 35.87%, 64.56%, 74.77%, and 102.54%; intercellular CO_2_ concentration (C_i_) by 9.44%, 15.19%, 16.42%, and 13.03%; and G_s_ by 31.66%, 66.40%, 83.52%, and 103.44%.

### 2.5. N Remobilization and Its Contributions

The results in Table 2 show that the total N remobilization was significantly regulated by nitrogen fertilizer application but was not affected by cover cropping. Under N-fertilized conditions, the N remobilization efficiency from stems-sheaths and leaves significantly decreased following the rotation with cover crops, with average reductions of 9.75% and 7.00%, respectively. The decrease was greater in the CC-Ry treatment than in the CC-Ra treatment. Compared to the CK treatment, both CC-Ra and CC-Ry treatments significantly reduced the contribution of total N remobilization to grains, with average decreases of 1.00% and 1.25%, respectively. Nitrogen application rate significantly increased the amount, rate, and grain contribution of plant N remobilization. Under CK conditions, N application increased the total N remobilization by an average of 81.41% relative to the N_0_ treatment. This increase primarily originated from leaves, where the N remobilization efficiency increased by an average of 22.50%, and its contribution to grain N increased by 8.00%. For stems-sheaths, the amount of N remobilization increased significantly by an average of 53.63%, the remobilization efficiency increased by an average of 6.25%, and its contribution to grain N increased by an average of 3.5%.

### 2.6. Principal Component Analysis

**Figure 5:**
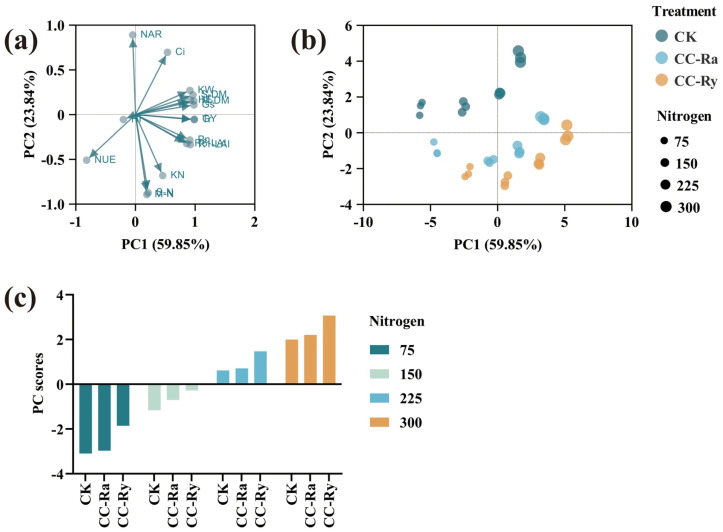
Principal component analysis of maize grain yield and grain yield-related factors; (**a**) represents the loading diagram of PCA; (**b**) represents the distribution of cover crops and nitrogen rate treatments in the PCA; (**c**) represents the PC scores of cover crops and nitrogen rate treatments. GY: grain yield, KN: kernel number per ha, HE: harvest ear per ha, KW: 1000-kernel weight, S-LAI: LAI at silking, M-LAI: LAI at maturity, NAR: net assimilation rate, S-DM: dry matter at silking, M-DM: dry matter at maturity, HI: harvest index, S-N: N content at silking, M-N: N content at maturity, NUE: N use efficiency, G_s_: stomatal conductance, P_n_: net photosynthetic rate, T_r_: transpiration rate, C_i_: intercellular CO_2_ concentration.

Principal component analysis (PCA) showed that grain yield and its related factors could be integrated into two principal components, PC1 and PC2. PC1 explained 59.85% of the total variation, and PC2 explained 23.84%, with a cumulative contribution rate of 83.96%. In the biplot analysis, grain yield was closely and positively correlated with DM, LAI, and net photosynthetic rate (P_n_), but was weakly correlated with leaf NAR, nitrogen accumulation, and HI. Yield was also negatively correlated with NUE, indicating that yield improvement may require a trade-off with NUE (Figure 5a). Figure 5b shows the distribution of cover crops and nitrogen (N) rate treatments in the PCA ordination. Cover crop incorporation increased PC1 scores but decreased PC2 scores, suggesting that cover crops promoted yield improvement while also enhancing NUE, although they were not conducive to increasing leaf NAR. In contrast, increasing the N rate increased both PC1 and PC2 scores, indicating that higher N application favored grain yield but was unfavorable for N accumulation and reduced NUE. Analysis of the comprehensive PCA scores (Figure 5c) showed that both cover crop rotation and increased N rate improved the overall performance. Specifically, compared with CK, CC-Ra and CC-Ry increased the comprehensive scores by an average of 0.22 and 1.01, respectively. Relative to N_75_, N_150_, N_225_, and N_300_ increased the comprehensive scores by an average of 1.92, 3.57, and 5.07, respectively.

## 3. Discussion

Rotational cover cropping is a cultivation practice in agroecology that was established as early as the last century for effectively improving soil quality [22] and increasing maize yield [18,23]. However, to this day, most maize-growing regions in China have not adopted rotational cover cropping as an essential production practice [24]. This is primarily attributed to the continuous advancement of nitrogen fertilizer production technology [25] and its significant contribution to ensuring food security [5,26]. Nevertheless, the long-term, heavy application of chemical fertilizers in croplands has placed an overwhelming burden on most of China’s land and ecological environments [2], leading to an increasing dependency of grain yield on fertilizer inputs. This vicious cycle has gradually garnered widespread attention from agricultural researchers [10]. Consequently, cover crops are re-entering the focus of Chinese scholars [27]. Understanding the yield-enhancing mechanisms of rotational cover cropping for maize and the challenges it faces constitutes an important theoretical foundation for its current popularization.

### 3.1. Effects of Cover Crops and Nitrogen Management on Maize Yield

Consistent with previous studies [19,28], the results of this research demonstrate that both cover crops promoted maize yield over the three-year experimental period. Notably, the yield levels under the CC-Ra and CC-Ry treatments at a nitrogen application rate of 225 kg ha^−1^ were comparable to that of the CK treatment under the N_300_ condition, indicating that cover crops can substitute for a portion of nitrogen fertilizer to stabilize maize yield [29,30]. The cover crops used in this study are both non-leguminous species. Their mechanisms of action primarily involve the incorporation of their own residues into the soil [29,31] and the improvement of soil structure and function through their root systems [32,33], while concurrently increasing the abundance of beneficial microorganisms in the soil [34,35], collectively influencing maize growth and development. Rye, characterized by a typical fibrous root system, distributes a large quantity of roots predominantly in the topsoil, effectively suppressing weed growth [17,18] and exhibiting a certain capacity to retain soil moisture and nutrients, thereby reducing soil erosion [36,37]. In comparison, using rapeseed as a rotational cover crop was less effective than using rye in enhancing maize yield. This is attributed to the stronger capacity of rye to conserve soil fertility and moisture after maize harvest [38]. However, some studies have reported that incorporating rye into rotations may not benefit maize yield [19] and can even lead to yield reduction [36,39]. This discrepancy arises from the different methods of integrating cover crops into the production system [40]. These methods not only create variations in available soil nutrients but also determine whether sufficient nutrients are supplied for the subsequent crop’s growth, as governed by the decomposition rate of the incorporated cover crop residues [41]. In the present study, the method of allowing cover crops to senesce naturally via frost and subsequently return to the soil [42] effectively conserved soil available nutrients, thereby laying the foundation for yield improvement in the following maize season [21]. Furthermore, during the 2024–2025 growing season, maize grain yields under the CC-Ra and CC-Ry treatments showed further increases compared to the N_225_ and N_300_ treatments. This suggests that integrating rotational cover crops with nitrogen management not only contributes to yield enhancement but also facilitates the full expression of the agronomic advantages inherent to cover crops within the rotation system [43,44].

### 3.2. Effects of Cover Crops and Nitrogen Application on Maize Dry Matter and Nitrogen Accumulation

The majority of previous research has primarily focused on the impacts of integrating cover crops into production systems on soil or the environment [20,37,45], with the study objects centered mainly on the cover crops themselves [17,21]. There has been a lack of in-depth analysis regarding the primary crop. Maize biomass and harvest index are key determinants of yield [46,47]. Our results indicate that cover crops contributed to yield increase predominantly by enhancing biomass, whereas the harvest index did not show a pronounced response (Figure 2a–c). Notably, the contribution of cover crops to maize biomass was greater under conditions of no or limited nitrogen fertilization (N_0_, N_75_) compared to treatments with sufficient nitrogen (N_225_, N_300_). This is likely because the stimulatory effect of nitrogen fertilizer application on maize biomass far exceeds that of cover crops [48]. It also suggests that rotational cover crops cannot entirely substitute for nitrogen fertilizer application; they require a combination with an appropriate nitrogen rate to fully realize their agronomic advantages [41].

Research has confirmed that cover crops play a significant role in the nitrogen cycle [49,50], particularly exhibiting a suppressive effect on greenhouse gases such as ammonia (NH_3_) and nitrous oxide (N_2_O) [14,18]. While this study did not systematically investigate the nitrogen cycle, both the CC-Ra and CC-Ry treatments significantly increased the nitrogen content of maize plants at the silking and maturity stages compared to the CK treatment (Figure 2d,e). Existing studies have verified that cover crops can enhance maize nitrogen content [28,30], suggesting that cover crops can mitigate a portion of nitrogen loss by promoting the growth and nutrient uptake of the primary crop [51]. Furthermore, the species of cover crops may also be a factor influencing nitrogen uptake by the primary crop [52], as evidenced by significant differences between the CC-Ra and CC-Ry treatments. Increasing the nitrogen application rate led to a significant decline in NUE [53]. Cover crops had a modest positive effect on NUE [54]. The likely reason is that NUE for each cropping pattern was calculated using its respective N_0_ treatment as the control. Since cover crops also promoted nitrogen uptake under zero-nitrogen conditions, this may have influenced the final calculation.

In conclusion, this study confirms that cover crops can improve NUE by influencing nitrogen uptake in the primary cash crop, which serves as key evidence for the role of cover crops in reducing nitrogen losses.

### 3.3. Maize Rotation with Cover Crops Increases the LAI While Decreasing the Net Assimilation Rate (NAR)

The LAI of maize is commonly used to represent the size of the canopy photosynthetic source [55,56]. In this study, both the inclusion of rotational cover crops and increased nitrogen application rate enhanced LAI (Figure 3a–e), which is likely a primary reason for the observed increase in maize biomass. This finding aligns with previous research [47,56]. Fitting the LAI data with quadratic functions revealed that the peak LAI values for both cover crop and nitrogen rate treatments occurred around 105–107 days after sowing (R1 stage). This indicates that the temporal pattern of maize leaf source development is largely unaffected by cover cropping or nitrogen application [48]. Gas exchange parameters of the ear leaf are key indicators of canopy photosynthetic capacity [57]. Compared to the CK treatment, both the CC-Ra and CC-Ry treatments significantly increased the net photosynthetic rate (P_n_). This enhancement is a direct consequence of increased nitrogen accumulation in the leaves, as an adequate nitrogen supply is crucial for supporting leaf photosynthetic activity [56]. It can be stated that cover crops not only increase the total leaf area per unit of land but also improve the photosynthetic efficiency of the leaves. However, analysis of the net assimilation rate (NAR) showed that rotational cover crops significantly reduced NAR. This suggests that cover cropping may lead to redundancy in leaf area [58]. Alternatively, it implies that the combination of cover crops and nitrogen fertilization can support a larger sink capacity. Since all experimental treatments in this study were conducted under the same planting density, this density likely imposed an upper limit on the potential influence of cover crops on the population sink size. Future studies are needed to further investigate this aspect.

### 3.4. Principal Component Analysis Elucidated the Comprehensive Impact of Maize Rotation with Cover Crops and Nitrogen Application on Yield

Principal component analysis (PCA) confirmed that integrating rotational cover crops with nitrogen fertilization significantly promotes yield [44]. However, this yield increase was accompanied by a significant decline in NUE [15]. This warns that agricultural production must balance the pursuit of high yield with fertilizer efficiency [2] and environmental protection [59]. Notably, the inclusion of rotational cover crops increased the comprehensive PCA score across all nitrogen rates. This indicates that introducing cover crops into maize production systems can enhance yield to a certain extent [44]. Simultaneously, observing the positions of different treatments in the two-dimensional distribution plot of principal components PC1 and PC2 reveals that while cover crops increase maize yield, they also facilitate nitrogen accumulation and improve NUE [28,48], albeit at the cost of reduced leaf NAR. This insight suggests that implementing rotational cover crops requires consideration of the balance between source and sink capacities in the main crop population [58], which is crucial for leveraging the full advantages of cover crops in production systems.

## 4. Materials and Methods

### 4.1. Site Description

This study was conducted at the Gongzhuling Experimental Station of Jilin Academy of Agricultural Science (43°52′ N, 124°81′ E, 202 m altitude) in Jilin Province, China, from 2021 to 2023. This area had a typical temperate continental monsoon climate, with a mean annual air temperature of 4.5 °C, a mean annual precipitation of 594.8 mm, an annual frost-free period of 133 days, an annual sunshine hours of 2712 h, and an annual effective accumulated temperature of 2800 °C. The soils at the study site were black soil with a soil texture of sandy clay loam, which can be classified as black soil (sand 31.04%, silt 51.77%and clay 17.19%). The initial soil profile (0–20 cm) contained 12.14 g kg^−1^, 1.21 g kg^−1^ TN, 0.45 g kg^−1^ TP, 20.12 g kg^−1^ TK, and 192.85 mg kg^−1^ available N, 38.97 mg kg^−1^ available P, 112 mg kg^−1^ available K, and had a pH value of 6.5 before the start of the experiment.

### 4.2. Experimental Design and Management

The field experiment used a three-year factorial study design with three replicates, considering three planting patterns (maize monocropping, CK; maize and rapeseed intercropping, CC-Ra; maize and cereal rye intercropping, CC-Ry) and five nitrogen (N) fertilizer application rates (0, 75, 150, 225, and 300 kg N ha^−1^). A total of 45 sample plots were split, and each plot was 7.8 × 5 m^2^. A common maize cultivar ‘Fumin 985’ in the region was planted annually at a density of 70,000 plants ha^−1^ in early May using a wide–narrow row cropping system (90 cm and 40 cm in row width, respectively). Two cover crop species with distinct root patterns, tap-rooted rapeseed (*Brassica napus* L., cultivar ‘Longyou 7’) and fibrous-rooted cereal rye (*Secale cereale* L., cultivar “BK-1”), were sown in late August. Cover crops were strip-sown in the middle of the 90 cm wide maize rows. The specific procedure was as follows: shallow furrows (3–5 cm deep) were opened manually within the wide maize rows. Rape and rye seeds were sown at seeding rates of 75 kg ha^−1^ and 150 kg ha^−1^, respectively. After sowing, the furrows were covered with soil, pressed firmly, and irrigated with a small amount of seedling-establishing water to ensure emergence. Both maize and cover crops were manually sown. In addition, maize straws were removed from the field after harvesting at the end of September each year. The two cover crop species failed to survive the winter, meaning they were terminated during the winter season. Before maize sowing in the following year, a small tillage machine was used to incorporate the cover crops into the soil by plowing, with simultaneous rotary tillage of the soil to a depth of 15 cm, and maize was transplanted in the seedbed of the strip-rotavated soil.

Fertilizer was applied in bands placed in furrows, and annually before the maize harvest. For N fertilizer application, urea (46% N) was applied as basal and topdressing fertilizer on the sowing day and at the V12 growth stage of maize growth, respectively. The ratio of basal and topdressing fertilizer remained constant at 1:1 across different N application rates. On the day of maize planting, 90 kg ha^−1^ of both P_2_O_5_ and K_2_O were also applied as basal fertilizer using superphosphate (P_2_O_5_, 44%) and potassium sulfate (K_2_O, 50%), respectively.

### 4.3. Measurements and Computations

#### 4.3.1. Grain Yield and Yield Component

At the maturity stage (R6), a sample segment consisting of the middle 4 rows with a length of 3 m per row was selected from each plot. All ears within the segment were harvested and weighed to calculate the average ear weight. Based on the average ear weight, 10 representative ears were selected from each treatment, air-dried for kernel trait analysis. The number of rows per ear and kernels per row were recorded to calculate the total number of kernels per ear. The grain moisture content and 100-kernel weight were determined using the oven-drying method, and the yield was converted to the standard yield at a moisture content of 14.0%.

Harvest index (HI) was calculated using the formula:

HI = Grain yield at R6/Dry matter at R6.

#### 4.3.2. Leaf Area and Dry Matter

At the flowering stage (Vt), three plants with consistent growth vigor were selected from each plot, and measurements were conducted every 10 days thereafter. The length (L) and maximum width (W) of each fully expanded leaf were recorded.

Leaf area was calculated using the formula:

LA (cm^2^) = L × W × 0.75

Leaf area index was calculated as follows:

LAI = LA/unit land area

Net assimilation rate (NAR) was calculated as follows [60]:

NAR (g cm^−2^ day ^−1^) = (W_2_ − W_1_) (lnLA_2_ − lnLA_1_)/(t_2_ − t_1_) (LA_1_ − LA_2_)

where W_1_ and W_2_ are dry matter at times t_1_ and t_2_, respectively.

#### 4.3.3. Nitrogen Content and Nitrogen Use Efficiency (NUE)

Maize plants were sampled at the Vt and R6 stages. Each plant was separated into four organs: stem, leaf, leaf sheath, and ear. The separated plant parts were oven-dried, ground into powder, and passed through a 0.25 mm sieve. The total nitrogen content of the plant samples was determined using a semi-automatic Kjeldahl nitrogen analyzer after digestion (CN61 M/KDY-9820, Beijing, China) with a mixture of H_2_SO_4_ and H_2_O_2_. The NUE was calculated as the agronomic nitrogen use efficiency, using the formula:

NUE = (Grain yield under nitrogen application − Grain yield under no nitrogen application)/Amount of nitrogen applied.

#### 4.3.4. Gas Exchange Parameters

At the Vt stage, 20 days after flowering, and 40 days after flowering, measurements were performed between 9:00 a.m. and 12:00 p.m. on clear and cloudless days. A portable photosynthesis system (Li-6400XT, Li Cor, Lincoln, NE, USA) was used to determine the net photosynthetic rate (P_n_), stomatal conductance (G_s_), intercellular CO_2_ concentration (C_i_), and transpiration rate (T_r_) of the ear leaf of maize. The measurement conditions were set as follows: photosynthetically active radiation (PAR) of 1800 μ mol m^−2^·s^−1^, CO_2_ concentration of 500 μ mol mol^−1^, and relative humidity of approximately 80%.

### 4.4. Statistical Analysis

Statistical analyses were conducted by using Origin 2022 (OriginLab, Northampton, MA, USA). The data sources were analyzed through a three-way ANOVA followed by the LSD test to compare the differences among the groups. Pearson correlation coefficients were calculated using SPSS v. 21.0 (IBM Inc., Armonk, NY, USA) to identify interrelationships among the measured parameters. Regression analysis was performed to examine the relationship between yield and nitrogen under different cropping systems. Principal component analysis (PCA) was conducted in this study to better understand the comprehensive impact of measurement indicators on grain yield and NUE.

## 5. Conclusions

In maize production, increased nitrogen application remains a primary cultural practice for yield enhancement. Rotating with cover crops can substitute for a portion of nitrogen fertilizer while maintaining yield. This is primarily because rotational cover crops increase the LAI and net photosynthetic rate, thereby laying the foundation for greater DM and yield in the maize canopy. Furthermore, the integration of cover crops contributes to greater N accumulation within maize plants, mitigates N losses from the soil system, and consequently improves NUE. It is noteworthy that although rotational cover crops contribute significantly to improving maize yield and NUE, it is essential to balance the source–sink characteristics of the maize population to avoid a loss of the benefits derived from cover cropping.

## Figures and Tables

**Table 1:** Analysis of variance for gas exchange parameters with respect to period, cropping systems, and nitrogen.

	P_n_	T_r_	C_i_	G_s_
Period (P)	**	**	**	**
Cropping Systems (CS)	**	**	**	**
Nitrogen (N)	**	**	**	**
P × CS	**	**	**	**
P × N	**	**	**	**
CS × N	**	**	**	**
P × CS × N	**	**	**	**

Note: P_n_ represents the net photosynthetic rate, T_r_ represents the transpiration rate, C_i_ represents the intercellular CO_2_ concentration, and G_s_ represents the stomatal conductance; **, significant at 0.01 levels.

**Table 2:** Maize vegetative organ nitrogen (N) remobilization and its contribution to grain N under different treatments in 2025.

Nitrogen	Cropping Systems	N Remobilization (g/plant)			N Remobilization Efficiency (%)		Contribution to Grain N by N Remobilization (%)		
		Stem-Sheath	Leaf	Total	Stem-Sheath	Leaf	Stem-Sheath	Leaf	Total
N_0_	CK	0.62 a	0.17 b	0.78 b	0.63 a	0.20 c	0.29 a	0.08 b	0.37 ab
	CC-Ra	0.50 b	0.40 a	0.90 a	0.48 b	0.41 a	0.21 b	0.17 a	0.39 a
	CC-Ry	0.50 b	0.41 a	0.91 a	0.41 c	0.37 b	0.19 b	0.16 a	0.35 b
N_75_	CK	0.93 a	0.49 a	1.41 a	0.68 a	0.45 a	0.33 a	0.18 a	0.51 a
	CC-Ra	0.93 a	0.47 a	1.41 a	0.61 b	0.39 b	0.32 ab	0.16 a	0.48 ab
	CC-Ry	0.95 a	0.48 a	1.43 a	0.56 c	0.35 c	0.31 b	0.16 a	0.46 b
N_150_	CK	0.91 a	0.44 a	1.35 a	0.67 a	0.39 a	0.29 a	0.14 a	0.43 a
	CC-Ra	0.91 a	0.43 a	1.34 a	0.59 b	0.33 b	0.27 ab	0.13 a	0.41 ab
	CC-Ry	0.90 a	0.44 a	1.34 a	0.52 c	0.30 c	0.26 b	0.12 a	0.38 b
N_225_	CK	1.15 a	0.55 a	1.70 a	0.75 a	0.47 a	0.38 a	0.18 a	0.57 a
	CC-Ra	1.16 a	0.56 a	1.72 a	0.70 b	0.42 b	0.37 a	0.18 ab	0.55 a
	CC-Ry	1.14 a	0.54 a	1.68 a	0.63 c	0.37 c	0.35 b	0.16 b	0.51 b
N_300_	CK	0.82 a	0.38 b	1.20 b	0.67 a	0.39 a	0.30 a	0.14 ab	0.45 a
	CC-Ra	0.80 a	0.37 b	1.18 b	0.59 b	0.33 b	0.29 a	0.13 b	0.42 a
	CC-Ry	0.86 a	0.46 a	1.32 a	0.56 c	0.35 b	0.29 a	0.15 a	0.44 a
ANOVA	Nitrogen (N)	**	**	**	**	**	**	**	**
	Cropping Systems (CS)	ns	**	ns	**	**	**	ns	**
	N × CS	ns	**	ns	**	**	**	**	ns

Note: CK represents the maize monocropping, CC-Ra represents the maize and rapeseed intercropping, and CC-Ry represents maize and cereal rye intercropping. Different small letters indicate significant differences under the same N level at *p* < 0.05 as determined by the LSD test; ns, not significant; **, significant at 0.01 levels.

## Data Availability

The raw data supporting the conclusions of this article will be made available by the authors on request.
